# Physicochemical Properties and Functional Characteristics of Ecologically Extracted Shrimp Chitosans with Different Organic Acids during Demineralization Step

**DOI:** 10.3390/molecules27238285

**Published:** 2022-11-28

**Authors:** Abir El-araby, Lahsen El Ghadraoui, Faouzi Errachidi

**Affiliations:** Functional Ecology and Environment Engineering Laboratory, Faculty of Science and Technology, Sidi Mohamed Ben Abdellah University, Imouzzer Street, B.P. 2202, Fez 30050, Morocco

**Keywords:** demineralization, organic acids, mineral acids, chitosan, degree of deacetylation, molecular weight, characterization

## Abstract

The current study aims to develop eco-friendly and economical chitosans with a wide range of applications using organic acids for shrimp shells demineralization. Chitosan samples were extracted from shrimp (*Parapenaeus longirostris*) shells and the demineralization step was performed with three organic acids (citric, acetic, and lactic) and two mineral acids (hydrochloric and sulfuric). The chitosans were characterized by Fourier transform infrared (FTIR) spectroscopy, X-ray diffraction (XRD), and scanning electron microscopy (SEM). The chitosans’ physicochemical properties were also determined. The characteristic bands and functional groups of the chitosans were identified by FTIR spectra. The chitosans’ crystallinity order was as follows: ***Ch_HCl_*** > ***Ch_Citric_*** > ***Ch_H2SO4_*** > ***Ch_Lactic_*** > ***Ch_Acetic_***. The chitosans’ morphological characteristics revealed a smooth surface and fibrous structures with pores. Chitosans extracted by organic acids showed the highest extraction yields. ***Ch_HCl_*** and ***Ch_Citric_*** had higher degrees of deacetylation values; 83.67% and 81.47%, respectively. The solubility was proportional to the degree of deacetylation. Furthermore, ***Ch_H2SO4_*** and ***Ch_Citric_*** had lower molecular weight values; 149 kDa and 183 kDa, respectively. Organic acids are as effective as mineral acids for shrimp shells demineralization. The developed process opens up possibilities to produce chitin and chitosan in a more eco-friendly way and at a lower cost in many industrial sectors.

## 1. Introduction

The shellfish processing industry produces several million tons of marine waste each year. These marine co-products, without any parallel development of added value, harm the environment by generating real pollution problems. The re-utilization of these natural resources is an important issue, as it constitutes an alternative method to minimize marine waste and respond to environmental problems. Chitin is mainly found in crustacean exoskeletons, insect cuticles, algae, and fungi cell walls [1,2]. This biopolymer is considered the second most abundant polysaccharide after cellulose, closely associated with proteins, minerals, lipids, and pigments. The term “chitosan” was given to deacetylated chitin by Hoppe-Seiler in 1894 [3]. Chitosan is a linear copolymer of D-glucosamine (2-amino-2-deoxy-D-glucose) and *N-*acetyl-D-glucosamine (*N*-acetyl-2-amino-2-deoxy-D-glucose) units with β-(1→4) linkages [4,5]. Usually, chitin and chitosan are obtained by three types of extraction processes: chemical, biological and enzymatic. The biological and the enzymatic methods require long processing times (several days). However, the chemical method requires a short processing time and can, therefore, be applied on an industrial scale. This technique can be modified to develop an environmentally friendly product with the most favorable physicochemical properties, allowing it to be used in a wide range of applications. Chitosan is currently attracting researchers’ attention due to its biodegradability [6], biocompatibility [7], non-toxic nature, unique physicochemical structure, and bioactive properties [8,9,10].

This versatile and eco-friendly polymer has a wide range of applications in agriculture, food industry, active packaging, pharmaceutical industry, textiles, biotechnology, and many other fields [11,12,13]. The spectrum of applications is mainly related to the physicochemical properties of the polymer. Chitosan is soluble in aqueous acids, such as acetic acid and lactic acid, and its solubility mainly depends on two key factors: the degree of deacetylation (DD) and the molecular weight (Mw); a high DD and low Mw increase the solubility [2,14]. The degree of deacetylation is a vital parameter because it influences the physical, chemical, mechanical, and biological properties of the biopolymer [15,16]. The molecular weight of chitosan is another significant parameter that affects its bioactive properties [17]. The -OH and -NH_2_ functional groups of chitosan allow the preparation of a variety of derivatives that improve its physicochemical properties, and at the same time, increase the spectrum of applications in various fields [18]. Chitosan derivatives have also been produced, with the aim of introducing new functions or properties.

The chemical extraction of chitosan from crustacean waste is a specific process that includes four steps: elimination of proteins (deproteinization), removal of pigments (discoloration), elimination of inorganic CaCO_3_ (demineralization), and converting chitin to chitosan (deacetylation). Deproteinization is usually performed by alkaline treatment, mainly with NaOH and KOH. This step depends on the alkali concentration, the temperature, and the solid–solvent ratio (*w*/*v*) [19]. Discoloration is an additional step that is only required if a colorless product is desired [20]. A mixture of organic solvents or acetone has been employed to remove pigments, such as carotenoids [21]. Demineralization consists of removing minerals by diluted acids. Hydrochloric acid (HCl) is the most commonly used [22,23]. The deacetylation step involves the removal of the acetyl group from the molecular chain of chitin. *N-*deacetylation of chitin is usually achieved by a concentrated alkali, such as NaOH or KOH (40–50%), at high temperatures [24]. Treatment with strong acids and bases during the demineralization and deproteinization processes is not environmentally friendly [25] and increases production costs.

Previous studies have revealed that the physicochemical and biological properties of chitosan are attributed to the origins of the polymer and the extraction process, including the three main steps: deproteinization, demineralization, and deacetylation. Many researchers have optimized the deacetylation conditions (NaOH concentration, temperature, and treatment time) to obtain chitosan with desirable properties applicable in several fields. To date, only a few studies have focused on the demineralization step. Today’s consumer is influenced by the organic trend. Therefore, our investigation aims to highlight the use of organic acids instead of mineral acids for the demineralization process and to study the feasibility of these organic acids appreciated by the food industry. The current study aims to develop ecological and economical chitosans with a wide range of applications using organic acids for the demineralization of shrimp shells. Chitosan samples were extracted from shrimp (*Parapenaeus longirostris*) shells waste and the demineralization step was performed with three organic acids (citric, acetic, and lactic) and two mineral acids (hydrochloric and sulfuric) in order to compare the final extraction products. The physicochemical properties of the chitosan samples, including ash content, moisture content, water binding capacity, fat binding capacity, solubility, degree of deacetylation, and average molecular weight, were determined. Furthermore, chitosan samples (extracted with organic and mineral acids) were characterized by Fourier transform infrared (FTIR) spectroscopy, X-ray diffraction (XRD), and scanning electron microscopy (SEM). The developed process opens up possibilities to produce chitin and chitosan in a more environmentally friendly way and at a lower cost in many industrial sectors.

## 2. Materials and Methods

### 2.1. Extraction of Chitosan Samples from Shrimp Shells

**Figure 1:**
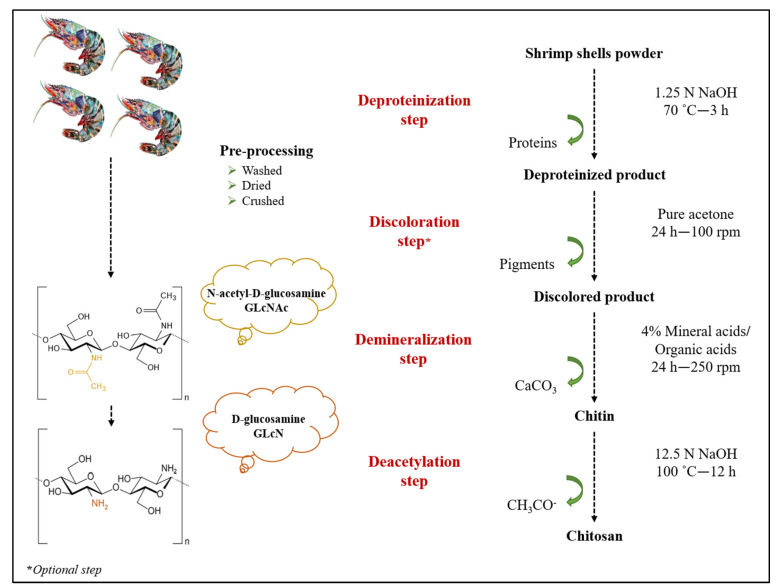
Chitosan extraction process from shrimp shells.

Chitosan extraction from shrimp shells involves three main steps: deproteinization, demineralization, and deacetylation. The discoloration is an additional step that is only required if a colorless product is desired. Shrimp (*Parapenaeus longirostris*) shells waste was obtained from a local fish market in Morocco. Shells were washed several times with warm distilled water to remove impurities, dried in an oven at 100 °C for 2 h, and then crushed into powder. The chitosan extraction process from shrimp shells waste is illustrated in Figure 1.

#### 2.1.1. Deproteinization Step

Shrimp shells powder was deproteinized by 1.25 N sodium hydroxide (NaOH) at a ratio of 1:8 (g/mL). The reaction was performed at 70 °C for 3 h. The product was collected, washed with distilled water until neutral pH, and then dried at 80 °C overnight to reach a constant weight. Afterward, discoloration was done with pure acetone at a ratio of 1:12 (g/mL) for 24 h under constant stirring (100 rpm). The resulting product was recovered, washed to neutrality, and then dried at 80 °C [26].

#### 2.1.2. Demineralization Step

This step was carried out with three organic acids (citric C₆H₈O₇, acetic CH₃COOH, and lactic C_3_H_6_O_3_) and two mineral acids (hydrochloric HCl and sulfuric H_2_SO_4_). Mineral acids were used as controls. Demineralization was performed using 4% from each acid at a ratio of 1:10 (g/mL) for 24 h at room temperature and under constant stirring (250 rpm). The obtained chitin was collected, washed with distilled water several times until neutral pH, and then dried at 80 °C.

#### 2.1.3. Deacetylation Step

Alkaline deacetylation is the process of transforming chitin into chitosan by removing acetyl groups. The purified chitin was deacetylated by 12.5 N sodium hydroxide (NaOH) at a ratio of 1:5 (g/mL). The deacetylation process was performed at 100 °C for 12 h. The chitosan obtained was filtered, washed with distilled water to neutrality, and completely dried at 80 °C [26].

In this manuscript, we noted the chitosan extracted with hydrochloric acid by (***Ch_HCl_***), the chitosan extracted with sulfuric acid by (***Ch_H2SO4_***), the chitosan extracted with citric acid by (***Ch_Citric_***), the chitosan extracted with acetic acid by (***Ch_Acetic_***), and the chitosan extracted with lactic acid by (***Ch_Lactic_***). All chitosan samples were weighed, and extraction yields (%) were determined.

### 2.2. Physicochemical Properties of Chitosan Samples

#### 2.2.1. Ash and Moisture Contents

The ash content was evaluated using 1 g of each chitosan sample into a muffle furnace at 650 °C for 3 h [27]. The ash values (%) are reported as the mean ± SD of three separate experiments.

The moisture content was determined by gravimetric analysis [28]. The samples were dried in an oven at 105 °C to constant weight [29]. The sample weights were measured after and before drying. Moisture content (%) was calculated according to Equation (1).

(1) $$\mathrm{Moisture}\;\left(\%\right)=\frac{\left(\mathrm{Initial}\;\mathrm{weight}\;\left(g\right)-\;\mathrm{Dry}\;\mathrm{weight}\;\left(g\right)\right)}{\left(\mathrm{Initial}\;\mathrm{weight}\;\left(g\right)\right)}\times 100$$

#### 2.2.2. Water Binding Capacity (WBC) and Fat Binding Capacity (FBC)

The water binding capacity (WBC) of ***Ch_HCl_***, ***Ch_H2SO4_***, ***Ch_Citric_***, ***Ch_Acetic_***, and ***Ch_Lactic_*** was performed by adding 0.5 g of each chitosan sample into initially weighed centrifuge tubes. Then, 10 mL of distilled water was added, and the contents were mixed with a vortex for 1 min to disperse the samples. The contents were left at room temperature for 30 min with intermittent shaking for 5 s every 10 min, then centrifuged at 3200 rpm for 25 min. After decanting the supernatants, the tubes were reweighed [30]. The WBC (%) was calculated using Equation (2).

(2) $$\mathrm{WBC}\;\left(\%\right)=\frac{\left(\mathrm{Water}\;\mathrm{bound}\left(g\right)\right)}{\left(\mathrm{Initial}\;\mathrm{sample}\;\mathrm{weight}\;\left(g\right)\right)}\times 100$$

The fat binding capacity (FBC) was measured by adopting a method similar to water binding capacity [30]. The 0.5 g of each chitosan sample was added into initially weighed centrifuge tubes. Then, 10 mL of soya oil was added, and the contents were mixed with a vortex for 1 min to disperse the samples. The contents were left at room temperature for 30 min with intermittent shaking for 5 s every 10 min, then centrifuged at 3200 rpm for 25 min. After decanting the supernatants, the tubes were reweighed. The FBC (%) was calculated using Equation (3).

(3) $$\mathrm{FBC}\;\left(\%\right)=\frac{\left(\mathrm{Fat}\;\mathrm{bound}\left(g\right)\right)}{\left(\mathrm{Initial}\;\mathrm{sample}\;\mathrm{weight}\;\left(g\right)\right)}\times 100$$

#### 2.2.3. Solubility Determination

The solubility test consists in dissolving 0.2 g of each chitosan sample (***Ch_HCl_***, ***Ch_H2SO4_***, ***Ch_Citric_***, ***Ch_Acetic_***, and ***Ch_Lactic_***) in 20 mL of 1% acetic acid at 60 °C under constant stirring for 24 h. Then, solutions were subjected to centrifugation (4000 rpm). The supernatant was removed, and the pellet (undissolved solid) was dried at 60 °C overnight. The insoluble content was determined in triplicate, and the solubility data of the chitosan samples were calculated using Equation (4).

(4) $$\mathrm{Solubility}\;\left(\%\right)=\frac{\left(W_{\mathrm{Initial}+S}-{\;W}_{\mathrm{Final}+S}\right)}{\left(W_{\mathrm{Initial}+S}-{\;W}_{\mathrm{Initial}}\right)}\times 100$$

W_Initial_ is the initial weight of tube;
W_Initial+S_ and W_Final+S_ are the initial and the final weight of tube + sample, respectively.

#### 2.2.4. Degree of Deacetylation (DD) Determination

The degrees of deacetylation (DD) of chitosan samples (***Ch_HCl_***, ***Ch_H2SO4_***, ***Ch_Citric_***, ***Ch_Acetic_***, and ***Ch_Lactic_***) were determined from FTIR spectra and by acid-base titration method. The chitosan samples were subjected to Fourier transform infrared (FTIR) spectroscopy. The degree of deacetylation (DD) was calculated from the FTIR spectra using Baxter’s formula Equation (5) [31].

(5) $$\mathrm{DD}\left(\%\right)=100-\left[\left(\frac{A_{1655}}{A_{3450}}\right)\times \left(\frac{100}{1.33}\right)\right]$$

A_1655_ represents absorption degree at 1655 cm^−1^;
A_3450_ represents absorption degree at 3450 cm^−1^.

The degree of deacetylation (DD) was measured by the acid-base titration method [32,33,34]. According to this method, 0.5 g of each chitosan samples were dissolved in 25.0 mL of 0.1 M HCl solution at room temperature under stirring. After that, 2 to 3 drops of 1% methyl orange indicator were added. Then, red solutions were titrated with 0.1 M NaOH solution until the color transitioned from red to yellow. The DD (%) was calculated using Equation (6).

(6) $$\mathrm{DD}\left(\%\right)=\frac{\left(C_{\mathrm{HCl}}\times {\;V}_{\mathrm{HCl}}-{\;C}_{\mathrm{NaOH}}\times {\;V}_{\mathrm{NaOH}}\right)}{G\;\times \left(100-\;W\right)\times 0.0994}\times 0.016$$

where, C**_HCl_** is the standard aqueous HCl solution concentration (M), C**_NaOH_** is the standard aqueous NaOH solution concentration (M), V_HCl_ is the standard aqueous HCl solution volume (mL), V_NaOH_ is the standard aqueous NaOH solution volume (mL), G is the chitosan samples weight (g), W is the water percentage of chitosan samples (%), 0.016 is the equivalent weight of amino groups (NH_2_) corresponding to 1 mL 0.1 M HCl (g), and 0.0994 is the theoretical equivalent mass of amino groups (NH_2_).

#### 2.2.5. Molecular Weight (Mw) Determination

The determination of the molecular weight (Mw) of chitosan samples (***Ch_HCl_***, ***Ch_H2SO4_***, ***Ch_Citric_***, ***Ch_Acetic_***, and ***Ch_Lactic_***) involves a rheological study with the aim of determining the intrinsic viscosity [η] essential for the determination of the Mw. Viscosity measurements were performed using an Ubbelohde capillary viscometer (f = 0.5 mm) at 25 ± 0.1 °C. Different dilute solutions of each chitosan sample were prepared (0.1%, 0.05%, 0.025%, and 0.0125%) and dissolved in 0.3 M HAc/0.2 M NaAc buffer [35]. The mean flow times were measured to obtain the intrinsic viscosity [η] values. The chitosan samples’ molecular weight (Mw) was determined by the Mark–Houwink–Sakurada formula, shown in Equation (7).

[η] = K · M_w_^α^ (7)

Constants K and α mainly depend on the nature of polymer and the solvent characteristics, as well as the temperature. In this study, the empirical constants were calculated (K = 9.55 × 10^−2^ and α = 0.73) with reference to previous work [36,37].

### 2.3. Characterization of Chitosan Samples

#### 2.3.1. Fourier Transform Infrared (FTIR) Spectroscopy

The extracted chitosan samples (***Ch_HCl_***, ***Ch_H2SO4_***, ***Ch_Citric_***, ***Ch_Acetic_***, and ***Ch_Lactic_***) were analyzed by Fourier transform infrared (FTIR) spectroscopy to determine the presence of the characteristic IR bands. Dried powdered samples were mixed with KBr and then pressed to create a homogenous sample/KBr disk. Chitosan samples infrared spectra were measured over the frequency range of 400 to 4000 cm^−1^ at a resolution of 4 cm^−1^ using a Bruker Vertex 70 spectrometer.

#### 2.3.2. X-ray Diffraction (XRD)

X-ray diffraction (XRD) analysis was performed to evaluate chitosan samples crystallinity by using an X’Pert-Pro diffractometer operating at 40 kV and 30 mA with Cu Kα radiation (1.54060 Å). Diffraction patterns were determined over a 2θ scan range of 5–90° in continuous mode. The Crystallinity Index (CrI) of chitosan samples was determined using Equation (8) proposed by [38].

(8) $$\mathrm{CrI}\;\left(\%\right)=\frac{\left[I_{110}-{\;I}_{\mathrm{am}}\right]\;}{I_{110\;}}\times 100$$

I_am_ is amorphous diffraction intensity at 2θ;
I_110_ is crystalline material maximum intensity at 2θ.

#### 2.3.3. Scanning Electron Microscopy (SEM) 

Scanning Electron Microscopy (SEM) was applied to examine the surface morphologies of chitosan samples (***Ch_HCl_***, ***Ch_H2SO4_***, ***Ch_Citric_***, ***Ch_Acetic_***, and ***Ch_Lactic_***). Surface images were evaluated by using a scanning electron microscope (JEOL JSM-IT500HR) performed at an accelerating voltage of 12 kV and a magnification range of ×1.000–15.000. Chitosan samples were coated with gold to acquire clear and accurate images.

### 2.4. Statistical Analyses

All the physicochemical analyses were performed in triplicates (*n* = 3), and the results are expressed as the mean values ± standard deviations. Data were analyzed using an analysis of variance (ANOVA) and Duncan’s multiple range test at *p* ≤ 0.05. The F value obtained was higher than the critical F′ value illustrated in the Fisher Snedecor table and therefore the difference is significant. The statistical analysis was developed with SPSS software. Principal component analysis (PCA) was done to identify the most significant basis for re-expressing the resulting data set and to correlate individuals (acids) with physicochemical characteristics.

## 3. Results and Discussion

### 3.1. Extraction of Chitosan Samples from Shrimp Shells

Chitosan samples (***Ch_HCl_***, ***Ch_H2SO4_***, ***Ch_Citric_***, ***Ch_Acetic_***, and ***Ch_Lactic_***) were extracted from shrimp (*Parapenaeus longirostris*) shells waste. Chitin/chitosan preparation from crustacean exoskeletons mainly involves three steps: deproteinization, demineralization, and deacetylation [39,40]. In this study, the demineralization was carried out with two mineral acids (hydrochloric and sulfuric) and three organic acids (citric, acetic and lactic). The mineral acids were used as controls. Shrimp shells demineralization by organic acids is as effective as mineral acids. The use of organic acids for shrimp shells demineralization is feasible and can lead to the development of biological demineralization process with organic acids, which is more environmentally adequate and can significantly reduce the production cost.

The yields of ***Ch_HCl_***, ***Ch_H2SO4_***, ***Ch_Citric_***, ***Ch_Acetic_***, and ***Ch_Lactic_*** were found to be 15.80%, 17.22%, 25.33%, 23.56%, and 24.47%, respectively. The chitosan samples extracted by organic acids showed the highest extraction yields due to the low degradation. A study by Yarnpakdee et al. (2022) reported that the yield of chitosan extracted from mantis shrimp (*Oratosquilla nepa*) shell decreased (14.13–15.79%) when the deacetylation time of chitin increased (2–4 h) [5]. The deacetylation conditions (NaOH concentration, temperature, and treatment time) are crucial in determining the yield of chitosan [41]. In our study, the extraction of the different chitosans was carried out from the same source and with the same extraction process. The only difference was the acid (organic or mineral) used during the demineralization of the shrimp shells. This allows us to suggest that the nature of the acid used for the demineralization step also has a direct impact on the extraction yield of the chitosans obtained. Olaosebikan et al. (2021) reported that the maximum yields of chitosan extracted from crab (*Callinectes amnicola*) shells and shrimp (*Penaeus notialis*) shells were 13.29% and 16.93%, respectively [42]. Chitosan was extracted from white-leg shrimp shells by chemical treatment, and the extraction yield was estimated to be 17.13 g/100 g of raw material weight [43]. The chitosan yield extracted from prawn shells was 22.08% [44]. It has been revealed in previous studies that these differences in chitosan extraction yield can be attributed to the origins of the polymer [45].

### 3.2. Physicochemical Properties of Chitosan Samples

#### 3.2.1. Ash and Moisture Contents

The ash and moisture contents of the chitosan samples are summarized in Table 1. Values (%) are reported as the mean ± SD of three separate experiments. Ash contents of ***Ch_HCl_***, ***Ch_H2SO4_***, ***Ch_Citric_***, ***Ch_Acetic_***, and ***Ch_Lactic_*** were measured as 0.05%, 0.03%, 0.02%, 0.19%, and 0.50%, respectively. From our results, the chitosan extracted by citric acid showed less ash content. Ash is an important indicator to evaluate the purity of chitosan and the efficiency of the demineralization process [46,47]. Furthermore, studies suggested that the ash content of high-quality chitosan should be <1% [48,49]. Kumari et al. (2017) reported that shrimp shells chitosan had less ash content, such as 0.03%, compared to fish and crab chitosan. The ash content of the chitosan primarily depends on the starting material and its composition [50].

The moisture contents of ***Ch_HCl_***, ***Ch_H2SO4_***, ***Ch_Citric_***, ***Ch_Acetic_***, and ***Ch_Lactic_*** were measured as 7.83%, 7.24%, 7.59%, 7.85%, and 7.52%, respectively. A study by Al-Manhel et al. (2018) revealed that chitosan prepared from shrimp shells had a moisture content of 7.84% [51]. Olaosebikan et al. (2021) reported that the moisture content of crab and shrimp shells was obtained as 20.2% and 25.5%, respectively [42]. It has been revealed in previous studies that the lower the moisture content of chitosan, the better its quality and shelf stability [44,52]. The moisture content of chitosan is largely related to the species of polymer [45].

#### 3.2.2. Water Binding Capacity (WBC) and Fat Binding Capacity (FBC)

The water binding capacity (WBC) and fat binding capacity (FBC) values of chitosan samples are summarized in Table 2. ***Ch_HCl_***, ***Ch_H2SO4_***, ***Ch_Citric_***, ***Ch_Acetic_***, and ***Ch_Lactic_*** exhibited water binding capacity values of 554%, 596%, 601%, 638%, and 587%, respectively. Cho et al. (1998) reported that the water binding capacity of shrimp shells chitosan samples ranged from 458% to 805% [53]. While the fat binding capacity values ranged from 314% to 535%. In our study, the fat binding capacity values of ***Ch_HCl_***, ***Ch_H2SO4_***, ***Ch_Citric_***, ***Ch_Acetic_***, and ***Ch_Lactic_*** were 429%, 442%, 437%, 384%, and 420%, respectively, though the water binding capacity and fat binding capacity mainly depend on the demineralization and deproteinization steps [28,54].

#### 3.2.3. Solubility Determination

Solubility is an important factor, especially for biopolymers applications. The solubility percentages of the chitosan samples were calculated, and the data obtained are summarized in Table 3. The solubility values of ***Ch_HCl_***, ***Ch_H2SO4_***, ***Ch_Citric_***, ***Ch_Acetic_***, and ***Ch_Lactic_*** were 78.45%, 70.22%, 74.18%, 69.02%, and 60.29%, respectively. Values are reported as the mean ± SD of three separate experiments.

The solubility of chitosan mainly depends on its biological species, molecular weight (Mw), and degree of deacetylation (DD) [2,18,55]. The solubility of chitosan in a 1% acetic acid aqueous solution can prove the success of polymer deacetylation [45]. The DD of chitosan, the ratio of *N*-acetyl-d-glucosamine to D-glucosamine structural units, impacts the polymer solubility in aqueous acidic solutions [56]. Depending on the production process and species used for the extraction of chitosan, a deacetylation degree of 85% is required for good solubility [4]. Kumari et al. (2017) reported that the solubility of chitosan extracted from fish scales, crab shells, and shrimp shells was found as 78%, 60%, and 70%, respectively [50]. In another study, chitosan extracted from blue crab (*Callinectes sapidus*) shells showed solubility of 94.15% [57]. Hossain and Iqbal (2014) produced chitosan from shrimp shell wastes and obtained the deacetylation degree and the solubility as 81.24% and 97.65%, respectively [28].

#### 3.2.4. Degree of Deacetylation (DD) Determination

The degree of deacetylation (DD) of chitosan samples (***Ch_HCl_***, ***Ch_H2SO4_***, ***Ch_Citric_***, ***Ch_Acetic_***, and ***Ch_Lactic_***) was determined from FTIR spectra and by acid-base titration method. The DD (%) obtained are summarized in Table 4. Values are reported as the mean ± SD of three separate experiments. The DD values of ***Ch_HCl_***, ***Ch_H2SO4_***, ***Ch_Citric_***, ***Ch_Acetic_***, and ***Ch_Lactic_*** determined from the FTIR spectra were 83.67%, 80.23%, 81.47%, 77.83%, and 69.14%, respectively. While the values of DD determined by the acid-base titration method were 85.61%, 79.84%, 85.20%, 78.64%, and 72.92% for ***Ch_HCl_***, ***Ch_H2SO4_***, ***Ch_Citric_***, ***Ch_Acetic_***, and ***Ch_Lactic_***, respectively. The degree of deacetylation is a vital parameter because it influences the physical, chemical, mechanical, and biological properties of the biopolymer [15,58]. The degree of deacetylation (DD) and the molecular weight (Mw) are the key factors characterizing chitosan by affecting its chemical and biological properties [18].

The correlation between the DD values evaluated from FTIR spectra and by acid-base titration method presents a linear model (FTIR = 0.9132 Titration + 8.7824) with a weighting coefficient (R^2^ = 0.8524). This confirms very well the quality of the two analyses (DD) performed. The degrees of deacetylation values determined from the FTIR spectra of chitosan extracted from shrimp and crab shells were calculated as 81.79% and 81.94%, respectively. While the DD values determined from the titration method were obtained as 84.76% and 82.15% for chitosan extracted from shrimp and crab shells, respectively [22]. The characterization of chitosan obtained from blue crab shells showed a degree of deacetylation value of 71% calculated by the FTIR spectra [57]. A study by [59] reported that chitosan samples were extracted from white shrimp shells using 40% (*w*/*v*) and 60% (*w*/*v*) NaOH in the deacetylation step and the deacetylation degrees were 94.23% and 92.45%, respectively. The DD of chitosan extracted from fungal (*Termitomyces titanicus*) biomass was calculated using conductimetric titration and was observed to be 69.50% [60].

It has been revealed in previous studies that these differences in the degree of deacetylation (DD) of chitosan can be attributed to the extraction process, as well as the initial source material [19,61]. Aldila et al. (2020) mentioned that the highest DD of chitosan was 88.89% when the deproteinization step was performed by 60% NaOH at 30 °C [62]. In the same report, chitosan DD increases while decreasing the deproteinization temperature and increasing NaOH concentration in the deacetylation step. Yarnpakdee et al. (2022) reported that the DD values of chitosan samples obtained from mantis shrimp (Oratosquilla nepa) by using various deacetylation times (2–4 h) ranged from 73.56% to 75.56% [5].

#### 3.2.5. Molecular Weight (Mw) Determination

The determination of the molecular weight Mw of chitosan samples extracted with different acids (***Ch_HCl_***, ***Ch_H2SO4_***, ***Ch_Citric_***, ***Ch_Acetic_***, and ***Ch_Lactic_***) was done by a mathematical calculation using the Mark–Houwink–Sakurada formula, shown in Equation (7). The intrinsic viscosity [η] of chitosan solutions in 0.3 M HAc/0.2 M NaAc at 25 °C and the corresponding molecular weight Mw are summarized in Table 5.

From Table 5, the chitosan samples extracted by mineral acids (***Ch_HCl_*** and ***Ch_H2SO4_***) exhibited molecular weight values of 229.184 kDa and 149.047 kDa, respectively. While the chitosan samples extracted by organic acids (***Ch_Citric_***, ***Ch_Acetic_***, and ***Ch_Lactic_***) had Mw values of 183.044 kDa, 224.317 kDa, and 255.248 kDa, respectively. The intrinsic viscosity [η] values are proportional to the molecular weight. Citric acid is a triacid, which explains its very important attack compared to monoacids (acetic and lactic acids). The same phenomenon occurs with mineral acids where sulfuric acid (diacid) showed a very important degradation compared to hydrochloric acid (monoacid). In addition to the strength of the acids, we note that the size of the used acids also affects the size of the elaborated chitosans, as the size of the acid increases, the size of the chitosan decreases. Studies on the intrinsic viscosity of chitosan show that the molecular weight (Mw), the degree of deacetylation (DD), and the distribution of acetyl groups are important parameters that significantly control the solubility of the biopolymer [46,63]. Various studies on chitosan indicate that low molecular weight chitosan, in the order of several tens of kDa, has significantly improved biological activities compared to high molecular weight polymer (ca 500 kDa) [64].

Acosta-Ferreira et al. (2020) mentioned that the chitosan obtained from shrimp shells had a low molecular weight (169 kDa) with 83% of DD [65]. In another study, chitosan extracted from shrimp shell waste by chemical treatment (deacetylation with 50% NaOH) had a molecular weight of 173 kDa [44]. The Mw of the chitosan and chitooligosaccharides extracted from white shrimp shells was 650 kDa and 13 kDa, respectively [43]. Molecular weight plays an important role in the rheological properties of biopolymers, such as chitosan, which directly impacts the development of chitosan-based biomaterials [66]. In addition, it has been reported that the intrinsic properties of chitosan, such as molecular weight (Mw) and degree of deacetylation (DD), could influence the performances of the chitosan films for food packaging [67].

### 3.3. Characterization of Chitosan Samples

#### 3.3.1. Fourier Transform Infrared (FTIR) Spectroscopy

**Figure 2:**
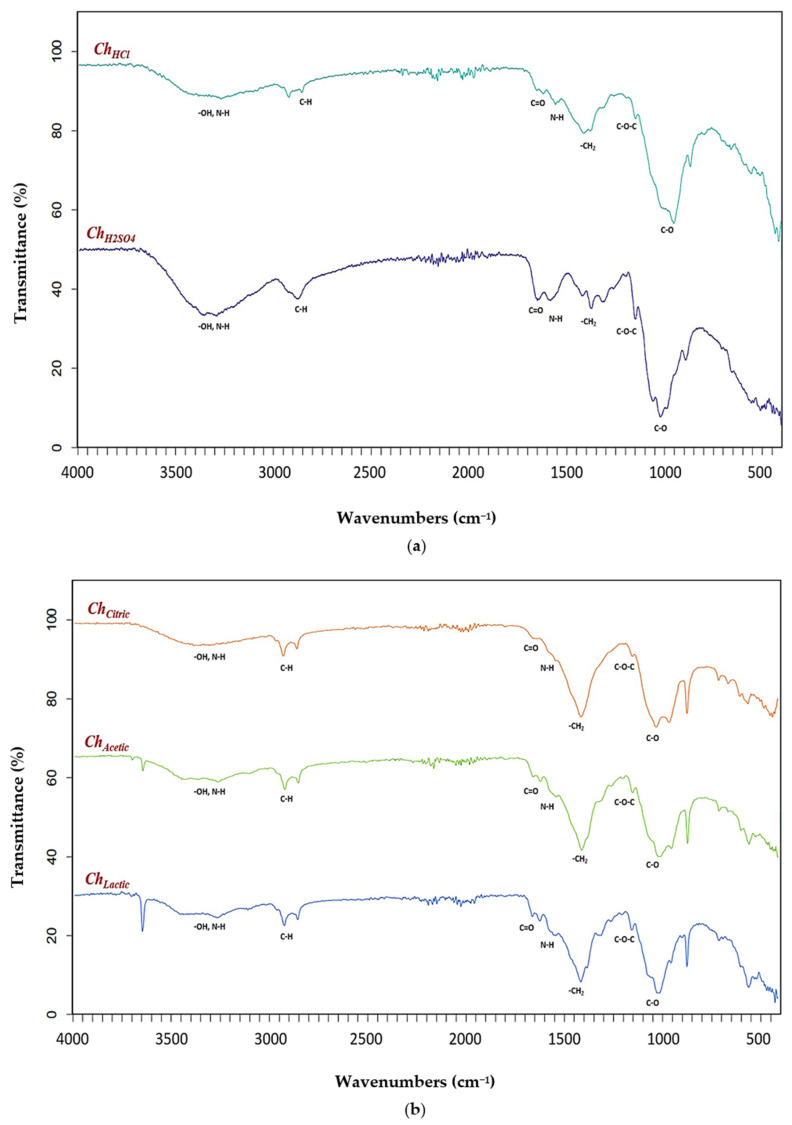
FTIR spectra of (**a**) chitosan samples extracted with mineral acids (***Ch_HCl_*** and ***Ch_H2SO4_***); (**b**) chitosan samples extracted with organic acids (***Ch_Citric_***, ***Ch_Acetic_***, and ***Ch_Lactic_***).

Functional groups in chitosan samples (***Ch_HCl_***, ***Ch_H2SO4_***, ***Ch_Citric_***, ***Ch_Acetic_***, and ***Ch_Lactic_***) were identified by Fourier transform infrared (FTIR) spectroscopy. The FTIR spectra obtained for chitosan samples extracted by mineral (hydrochloric and sulfuric) and organic (citric, acetic, and lactic) acids are shown in Figure 2.

The characteristic bands of chitosan samples (***Ch_HCl_***, ***Ch_H2SO4_***, ***Ch_Citric_***, ***Ch_Acetic_***, and ***Ch_Lactic_***) from spectral data were summarized in Table 6. The FTIR peaks for ***Ch_HCl_*** were observed at 3357 cm^−1^ (N-H and O-H stretching vibrations), 2920 cm^−1^ and 2877 cm^−1^ (axial stretching of C-H in the polymer chain), 1656 cm^−1^ (amide I vibration modes), 1568 cm^−1^ (N-H straining vibrations of NH_2_ groups), 1419 cm^−1^ (CH_2_ deformation vibrations), 1317 cm^−1^ and 1259 cm^−1^ (amide III vibration modes), 1149 cm^−1^ and 1060 cm^−1^ (C-O-C bridge), 1026 cm^−1^ and 985 cm^−1^ (C-O stretching vibration of alcohol groups). The FTIR peaks for ***Ch_H2SO4_*** were observed at 3328 cm^−1^ (axial stretching of hydroxyl groups (O-H) overlapped to the asymmetric/symmetric stretching of the amine bonds (N-H)), 2918 cm^−1^ and 2871 cm^−1^ ( C-H aliphatic stretching), 1652 cm^−1^ (C=O stretching of the acetamide groups of the chitosan), 1587 cm^−1^ (trans-secondary amides), 1377 cm^−1^ (symmetric deformation of C-H in the -CH_3_ group), 1318 cm^−1^ and 1256 cm^−1^ (-CO and NH_2_ of the amide III), 1151 cm^−1^ and 1064 cm^−1^ (glycosidic linkage C-O-C). The assignment of the characteristic bands of the saccharide structure of chitosans extracted with mineral acids (***Ch_HCl_***, ***Ch_H2SO4_*)** is based on the literature data [68,69].

The characteristic absorption bands of ***Ch_Citric_*** were exhibited at 3263 cm^−1^ (O-H and N-H stretching), 2920 cm^−1^ and 2885 cm^−1^ (CH-stretching), 1649 cm^−1^ (Amide I vibration), 1556 cm^−1^ (-NH_2_ bending), 1303 cm^−1^ and 1256 cm^−1^ (Amide III vibrations), 1253 cm^−1^ and 1062 cm^−1^ (anti-symmetric stretching of the C-O-C bridge), 1018 cm^−1^ and 985 cm^−1^ (skeletal vibrations involving the C-O stretching). The FTIR peaks for ***Ch_Acetic_*** were observed at 3421 cm^−1^ (O-H and N-H stretching vibration), 2921 cm^−1^ and 2870 cm^−1^ (CH_3_ symmetrical stretch), 1620 cm^−1^ (C=O secondary amide stretch), 1558 cm^−1^ (N-H bend and C-N stretch), 1425 cm^−1^ (CH_2_ ending and CH_3_ deformation), 1317 cm^−1^ and 1258 cm^−1^ (CH_2_ wagging of amide III), 1153 cm^−1^ and 1066 cm^−1^ (C-O-C asymmetric stretch in phase ring), 1021 cm^−1^ and 985 cm^−1^ (C-O asymmetric stretching). The spectra of ***Ch_Lactic_*** showed bands at 3358 cm^−1^ (hydroxyl groups (O-H) stretching overlapped to amine bonds (N-H) stretching), 2919 cm^−1^ and 2869 cm^−1^ (CH_2_ stretching), 1658 cm^−1^ (vibrational mode of amide C=O), 1584 cm^−1^ (trans-secondary amides), 1298 cm^−1^ and 1256 cm^−1^ (amide III vibration modes), 1163 cm^−1^ and 1062 cm^−1^ (C-O-C bridge stretching), 1004 cm^−1^ and 968 cm^−1^ (C-O stretching vibration in secondary and primary OH groups). The FTIR spectra of chitosan samples extracted with organic acids (***Ch_Citric_***, ***Ch_Acetic_***, and ***Ch_Lactic_***) were very similar to the FTIR results of chitosans extracted with mineral acids (***Ch_HCl_*** and ***Ch_H2SO4_***). From the analysis of Fourier transform infrared spectroscopy, we find that the use of organic acids for demineralization of shrimp shell to obtain chitosan is feasible, and this allows the idea of using organic acids instead of mineral acids for environmentally friendly extraction of chitin/chitosan.

Similar results have been obtained in previous studies. The bands observed at 3289 cm^−1^ and 2873 cm^−1^ were determined by the -OH stretching and C-H aliphatic stretching, respectively [70]. Amide I vibrational mode (C=O stretching) was observed at 1604 cm^−1^, 1598 cm^−1^, and 1592 cm^−1^ [71]. Absorption band at 1568 cm^−1^ was assigned to trans-secondary amides (amide II) [65]. Bands at 1072 cm^−1^ and 2879 cm^−1^ were attributed to C-O and C-H stretching vibrations, respectively [45]. Chitosan and chitooligosaccharides IR spectra revealed peaks around 1000–1200 cm^−1^, which were attributed to the polymer structure due to C-O-C bridge stretching [43,72]. Chitosan exhibits a distinctive band at 1594 cm^−1^ agreed with vibrations of amide III [73]. The peak at 1025 cm^−1^ corresponded to C-O stretching vibration of chitosan [70].

#### 3.3.2. X-ray Diffraction (XRD)

**Figure 3:**
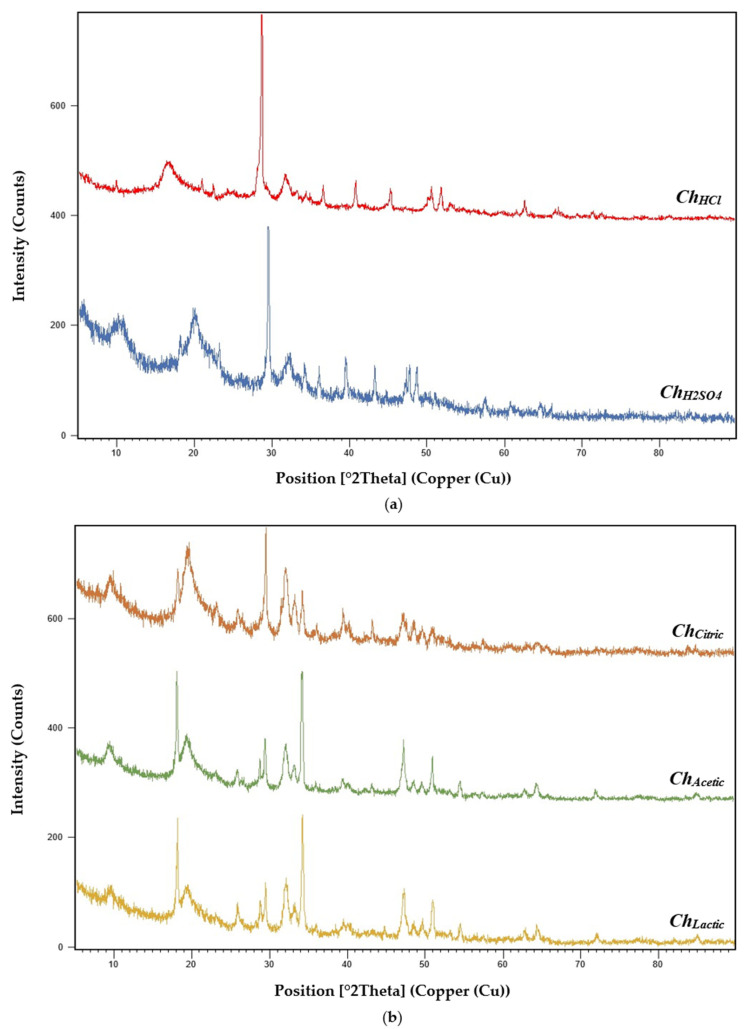
X-ray diffraction (XRD) pattern of (**a**) chitosan samples extracted with mineral acids (***Ch_HCl_*** and ***Ch_H2SO4_***); (**b**) chitosan samples extracted with organic acids (***Ch_Citric_***, ***Ch_Acetic_***, and ***Ch_Lactic_***).

The chitosan samples (***Ch_HCl_***, ***Ch_H2SO4_***, ***Ch_Citric_***, ***Ch_Acetic_***, and ***Ch_Lactic_***) were characterized by the X-ray diffraction technique (Figure 3). The XRD studies of ***Ch_HCl_*** showed a sharper peak at 20° (290.36 counts/s) and a maximum peak at 30° (640.09 counts/s). ***Ch_H2SO4_*** revealed stronger peaks at 10° (187.10 counts/s) and 20° (177.09 counts/s) and a maximum reflection at 30° (580.29 counts/s). ***Ch_Citric_*** XRD pattern showed the sharpest peak at 20° (169.29 counts/s) and the highest peak at 30° (340.37 counts/s). ***Ch_Acetic_*** and ***Ch_Lactic_*** XRD analysis revealed sharper peaks at 19° (471.59 counts/s and 415.96 counts/s, respectively) and maximum peaks at 35° (583.26 counts/s and 479.46 counts/s, respectively).

The XRD results of the chitosan samples showed the crystalline nature of obtained polymers. Similar results have been obtained in previous studies. El-araby et al. (2022) reported that the X-ray diffraction pattern of chitosan extracted from shrimp shells revealed two characteristic diffraction peaks at 2θ around 20° and 30°, which are typical fingerprints of crystalline chitosan character [26]. Another study reported that XRD studies of chitosan showed the maximum peak at 20.04° (113.92 counts/s), corresponding to chitosan crystalline nature [71]. X-ray diffraction (XRD) analysis was applied to detect obtained chitosan crystallinity [74]. The strongest sharp reflection was observed at 2θ around 30–35° (625 count/s). Chitosan extracted from Pacific white shrimp shells exhibited two peaks of crystalline character approximately at 10° and 20° (2θ) [59]. House cricket and commercial shrimp chitosan were analyzed by X-ray diffraction and nine diffraction peaks were observed at different values of 2θ, with three strong peaks at 9.6°, 19.6°, and 21.2° [75].

The Crystallinity Index (%) of chitosan samples extracted by mineral acids and organic acids (***Ch_HCl_***, ***Ch_H2SO4_***, ***Ch_Citric_***, ***Ch_Acetic_***, and ***Ch_Lactic_***) were determined using Equation (8), proposed by [38] (Table 7). The Crystallinity Index was calculated based on the intensity of the crystalline and amorphous regions [76].

In this study, chitosan samples extracted by mineral acids (***Ch_HCl_*** and ***Ch_H2SO4_***) show crystallinity index values of 87.43% and 82.61%, respectively. While chitosan samples extracted by organic acids (***Ch_Citric_***, ***Ch_Acetic_***, and ***Ch_Lactic_***) have crystallinity index values of 84.51%, 82.02%, and 82.24%, respectively. A study done by Ibitoye et al. (2018) in Malaysia, reported that the house crickets chitosan and commercial shrimp chitosan showed CrI values of 86.64% and 94.42%, respectively [75]. Kumari et al. (2017) mentioned that the CrI (%) values of commercial, shrimp, crab, and fish chitosan were 96%, 82%, 88%, and 84%, respectively [50]. The crystallinity index (CrI) value of the chitosan produced by the deacetylation of extracted chitins isolated from shrimp shells was 65% [25]. It has been revealed in previous studies that chitin/chitosane properties are principally depended on the natural origin and the extraction process [19]. X-ray diffraction analysis was performed to evaluate the crystallinity of the chitosan samples extracted by two mineral acids and three organic acids. The crystallinity order of the five chitosan samples is as follows: ***Ch_HCl_*** > ***Ch_Citric_*** > ***Ch_H2SO4_*** > ***Ch_Lactic_*** > ***Ch_Acetic_***.

#### 3.3.3. Scanning Electron Microscopy (SEM)

**Figure 4:**
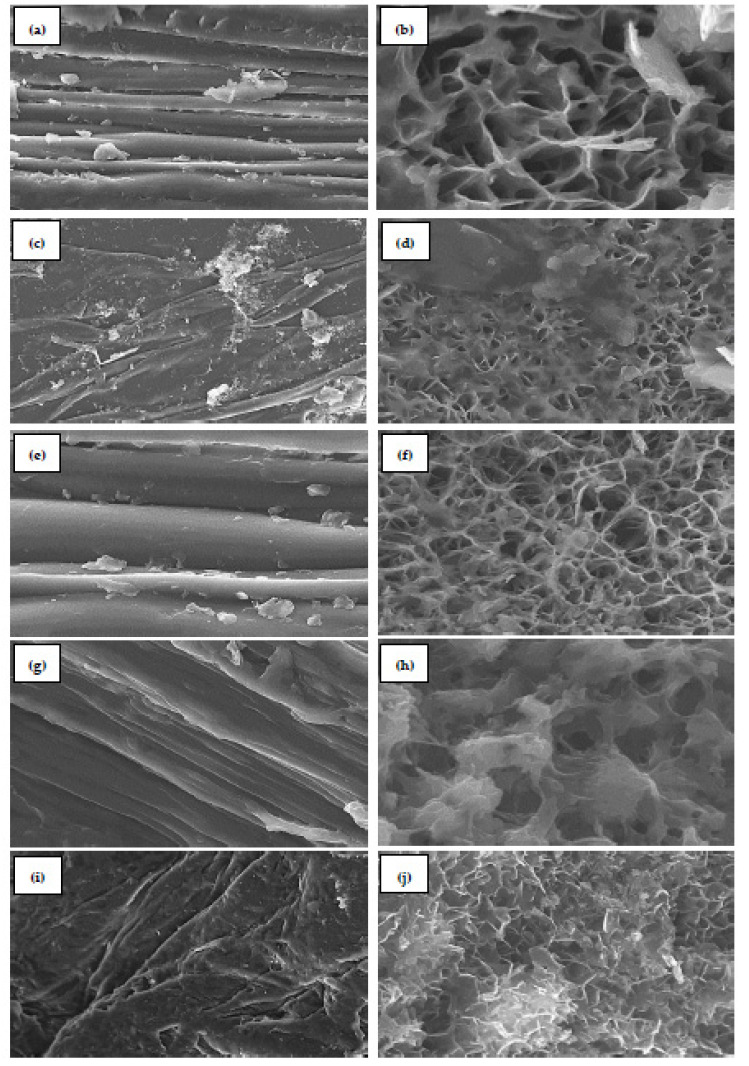
SEM images of (**a**,**b**) chitosan extracted with hydrochloric acid (***Ch_HCl_***) at 10µm and 1µm, respectively; (**c**,**d**) chitosan extracted with sulfuric acid (***Ch_H2SO4_***) at 10µm and 5µm, respectively; (**e**,**f**) chitosan extracted with citric acid (***Ch_Citric_***) at 5µm and 1µm, respectively; (**g**,**h**) chitosan extracted with acetic acid (***Ch_Acetic_***) at 5µm and 1µm, respectively; (**i**,**j**) chitosan extracted with lactic acid (***Ch_Lactic_***) at 10µm and 2µm, respectively.

The morphological characteristics of chitosan samples (***Ch_HCl_***, ***Ch_H2SO4_***, ***Ch_Citric_***, ***Ch_Acetic_***, and ***Ch_Lactic_***) were studied by scanning electron microscopy (Figure 4). ***Ch_HCl_*** presents a smooth surface morphology at 10 µm (Figure 4a) and fibrous structures with pores at 1 µm (Figure 4b). The overall surface morphology of ***Ch_H2SO4_*** shows a combination of rough and smooth layers at 10 µm (Figure 4c) and a combination of fibers and pores at 5 µm (Figure 4d). ***Ch_Citric_*** presents a very smooth surface at 5 µm (Figure 4e). Figure 4f reveals a porous and fibrous surface morphology at 1 µm. The nano-fiber structures are seen with a fractured appearance. The smooth top surface of ***Ch_Acetic_*** is observed at 5 µm in Figure 4g. Additionally, at 1 µm only big pores are observed in some areas (Figure 4h). ***Ch_Lactic_*** presents a hard and rough surface morphology at 10 µm (Figure 4i) and fibrous structures at 2 µm (Figure 4j).

The presence of fibers and pores combined in this study is in agreement with previous studies on chitin and chitosan from different sources [75]. Scanning electron microscopy (SEM) shows that chitosan produced from shrimp shells had a smooth and homogenous surface [25]. A study done by El-araby et al. (2022) reported that the micrographs of shrimp shell chitosan showed a relatively smooth top surface and fibrous structures [26].

### 3.4. Dimensional Analysis of the Resulting Data

**Figure 5:**
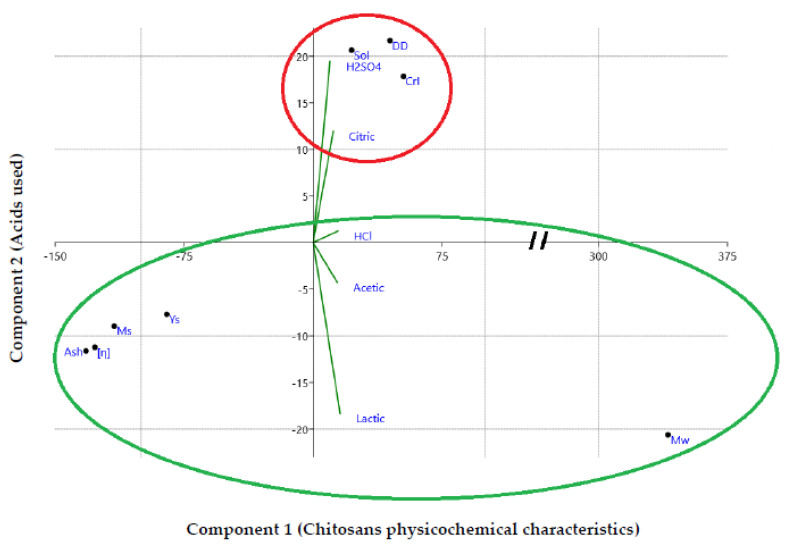
Dimensional analysis of the acids used (axis 1) and the physicochemical characteristics of the chitosans developed (axis 2).

The physicochemical properties of the extracted chitosans (***Ch_HCl_***, ***Ch_H2SO4_***, ***Ch_Citric_***, ***Ch_Acetic_***, and ***Ch_Lactic_***) were subjected to a dimensional analysis (Principal Component Analysis) (Figure 5) in order to identify the qualitative (nature of the acid used) and quantitative (physicochemical properties) variables involved in the development of ecological and economical products with a wide range of applications.

The first synthetic component (axis 1) and the second synthetic component (axis 2) correspond to the physicochemical characteristics of chitosans and acids used during the demineralization step, respectively. Figure 5 shows that solubility (Sol), degree of deacetylation (DD), and crystallinity index (CrI) contribute to the positive part of components 1 and 2, while molecular weight (Mw) contributes to the positive part of component 1, only. Yields (Ys), moisture content (Ms), ash content, and intrinsic viscosity [η] contribute to the negative part of the component 1 and 2. H_2_SO_4_, citric, and HCl acids contribute to the positive part of component 2, while acetic and lactic acids contribute to its negative part. The most important parameters affecting chitosan applications vary according to the acids (organic or mineral) used in the demineralization step. Acetic and lactic acids were positively correlated with molecular weight (Mw) of extracted chitosans. While H_2_SO_4_, citric, and HCl acids were positively correlated with solubility, degree of deacetylation, and crystallinity index. ***Ch_Lactic_*** exhibited the highest molecular weight value and the lowest degree of deacetylation value (255 kDa and 69.29%, respectively). The DD and Mw of ***Ch_Acetic_*** were calculated as 77.83% and 224 kDa, respectively. ***Ch_H2SO4_*** and ***Ch_Citric_*** had lowest molecular weight values (149 kDa and 183 kDa, respectively). The degree of deacetylation of ***Ch_H2SO4_*** and ***Ch_Citric_*** were calculated as 80.23% and 81.47%, respectively. The DD and Mw of ***Ch_HCl_*** were calculated as 83.67% and 229 kDa, respectively. The degrees of deacetylation values are proportional to the chitosans’ solubility. ***Ch_Citric_*** showed a crystallinity index value of 84.51%. From our results, we can conclude that citric acid is as effective as sulfuric acid for the demineralization of shrimp shells. ***Ch_Citric_*** revealed the most favorable physicochemical properties, which allows it to be used in a wide range of food and biomedical applications. High MW chitosan led to the improvement of rice shoot and root growth under drought stress [77]. However, the low solubility and high viscosity of chitosan at high Mw make it difficult to use, and thus, limit its industrial applications [78].

Chitosan properties depend on several important factors, such as the degree of deacetylation (DD), molecular weight (Mw), and crystallinity [66]. Chitosan solubility mainly depends on two key factors: degree of deacetylation and molecular weight; a high DD and low Mw increase the solubility [2,14,63]. The chitosan crystallinity increases as its degree of deacetylation increases [16]. The degree of deacetylation and the molecular weight of chitosan affect the physical, chemical, mechanical, and biological properties of the biopolymer [15,16,17]. Chitosan with a lower molecular weight (MW) and a higher degree of deacetylation (DD) revealed stronger bioactivities [40]. In many works, it has been reported that molecular weight enhances chitosan antimicrobial (antibacterial and antifungal) activities [18]. Chitosan molecular weight has been shown to affect oxidative properties. The antioxidant activity of chitosan increases as its molecular weight decreases [79]. The applications of chitosan are generally a function of deacetylation degree and molecular weight. In previous studies, low molecular weight chitosan has been used as a food additive and as an antimicrobial packaging material to improve food safety and shelf life [78,80]. Deacetylation degree and molecular weight could influence the performances of the chitosan films for food packaging [67]. Various studies done on chitosan indicate that low molecular weight chitosans have significantly improved biological activities when compared to high molecular weight polymers (ca 500 kDa) [64]. Low molecular weight chitosan (50–190 kDa) [81] has been highlighted in a wide range of food and biomedical applications [66].

## 4. Conclusions

The development of an eco-friendly chitin demineralization process that is less costly when compared to conventional ones, is less harmful to ecosystems, and shows promise for industrial applications is desirable. The current study aims to highlight the use of organic acids instead of mineral acids for the demineralization process and to investigate the feasibility of chitosan extraction by these organic acids. Citric, acetic, and lactic acids are as effective as hydrochloric and sulfuric acids (used as controls) for shrimp shells demineralization. This diversity of acids used during the demineralization of shrimp shells could be advantageous insofar as we can elaborate a range of chitosans that are usable in several disciplines. The nature of the acid used during the demineralization step of chitosans affects their physicochemical properties, and consequently, their biological activities. Chitosan extracted with citric acid revealed the most favorable physicochemical properties, 183 kDa and 81.47% for molecular weight and degree of deacetylation, respectively, allowing it to be used in a wide range of food and biomedical applications. Our contribution is double-edged, eliminating toxic acids (mineral acids) for public health and protecting the environment by recovering marine waste.

## Figures and Tables

**Table 1:** Ash and moisture contents (%) of chitosans extracted with mineral acids (***Ch_HCl_*** and ***Ch_H2SO4_***) and organic acids (***Ch_Citric_***, ***Ch_Acetic_***, and ***Ch_Lactic_***).

Chitosan Samples	Mineral Acids		Organic Acids		
	*Ch_HCl_*	*Ch_H2SO4_*	*Ch_Citric_*	*Ch_Acetic_*	*Ch_Lactic_*
Ash	0.05 ± 0.10 ^b^	0.03 ± 0.02 ^a^	0.02 ± 0.07 ^a^	0.19 ± 0.04 ^c^	0.50 ± 0.17 ^d^
Moisture	7.83 ± 0.20 ^d^	7.24 ± 0.06 ^a^	7.59 ± 0.09 ^c^	7.85 ± 1.00 ^d^	7.52 ± 1.02 ^b^

The values followed by the same letter a–d in the same line show no statistically significant differences (*p* < 0.05). Mean values (*n* = 3) ± standard error.

**Table 2:** Water binding capacity (WBC) and fat binding capacity (FBC) of chitosans extracted with mineral acids (***Ch_HCl_*** and ***Ch_H2SO4_***) and organic acids (***Ch_Citric_***, ***Ch_Acetic_***, and ***Ch_Lactic_***).

Chitosan Samples	Mineral Acids		Organic Acids		
	*Ch_HCl_*	*Ch_H2SO4_*	*Ch_Citric_*	*Ch_Acetic_*	*Ch_Lactic_*
WBC (%)	554 ± 11.3 ^a^	596 ± 09.8 ^c^	601 ± 14.6 ^c^	638 ± 11.0 ^d^	587 ± 13.8 ^b^
FBC (%)	429 ± 21.0 ^c^	442 ± 16.5 ^e^	437 ± 10.8 ^d^	384 ± 11.4 ^a^	420 ± 17.2 ^b^

The values followed by the same letter a–e in the same line show no statistically significant differences (*p* < 0.05). Mean values (*n* = 3) ± standard error.

**Table 3:** Solubility (%) of chitosans extracted with mineral acids (***Ch_HCl_*** and ***Ch_H2SO4_***) and organic acids (***Ch_Citric_***, ***Ch_Acetic_***, and ***Ch_Lactic_***).

Chitosan Samples	Mineral Acids		Organic Acids		
	*Ch_HCl_*	*Ch_H2SO4_*	*Ch_Citric_*	*Ch_Acetic_*	*Ch_Lactic_*
Solubility	78.45 ± 0.10 ^d^	70.22 ± 0.03 ^b^	74.18 ± 0.20 ^c^	69.02 ± 0.01 ^b^	60.29 ± 0.02 ^a^

The values followed by the same letter a–d in the same line show no statistically significant differences (*p* < 0.05). Mean values (*n* = 3) ± standard error.

**Table 4:** Degree of deacetylation (%) of chitosan samples obtained from FTIR spectra and by the acid-base titration method.

Chitosan Samples	Mineral Acids		Organic Acids		
	*Ch_HCl_*	*Ch_H2SO4_*	*Ch_Citric_*	*Ch_Acetic_*	*Ch_Lactic_*
DD _FTIR_	83.67 ± 0.6 ^e^	80.23 ± 0.0 ^c^	81.47 ± 0.4 ^d^	77.83 ± 0.3 ^b^	69.14 ± 1.2 ^a^
DD _Titration_	85.61 ± 0.2 ^c^	79.84 ± 0.6 ^b^	85.20 ± 0.9 ^c^	78.64 ± 1.0 ^b^	72.92 ± 0.0 ^a^

The values followed by the same letter a–e in the same line show no statistically significant differences (*p* < 0.05). Mean values (*n* = 3) ± standard error.

**Table 5:** Intrinsic viscosity [η] and molecular weight Mw of chitosans extracted with mineral acids (***Ch_HCl_*** and ***Ch_H2SO4_***) and organic acids (***Ch_Citric_***, ***Ch_Acetic_***, and ***Ch_Lactic_***).

Chitosan Samples	Mineral Acids		Organic Acids		
	*Ch_HCl_*	*Ch_H2SO4_*	*Ch_Citric_*	*Ch_Acetic_*	*Ch_Lactic_*
[η] (dL/g)	2.6943	1.9179	2.2559	2.6490	2.9336
Mw (kDa)	229.184	149.047	183.044	224.317	255.248

**Table 6:** The FTIR bands (cm^−1^) of chitosans extracted with mineral acids (***Ch_HCl_*** and ***Ch_H2SO4_***) and organic acids (***Ch_Citric_***, ***Ch_Acetic_***, and ***Ch_Lactic_***).

Functional Groups and Vibration Modes	*Ch_HCl_*	*Ch_H2SO4_*	*Ch_Citric_*	*Ch_Acetic_*	*Ch_Lactic_*
Stretching vibrations of hydroxyl groups (OH) Asymmetric/symmetric stretching of the amine bonds (NH_2_)	3357.91	3328.98	3263.40	3421.55	3358.63
C-H aliphatic stretching vibration (CH_2_)	2920.09 2877.65	2918.16 2871.87	2920.38 2885.37	2921.59 2870.21	2919.25 2869.94
Amide frequencies of C=O bond stretching of amide I	1656.77	1652.91	1649.70	1620.63	1658.13
N-H bending vibrations of NH_2_ groups of the amide II	1568.05	1587.34	1556.48	1558.41	1584.41
CH_2_ deformation vibrations in the CH_2_OH groups	1419.54	1421.47	1431.11	1425.32	1404.11
Symmetrical angular deformation of CH_3_ in NHCOCH_3_ groups	1375.18	1377.11	1380.96	1380.43	1369.39
C-N stretching vibrations of amide III	1317.31 1259.45	1318.24 1256.60	1303.81 1256.60	1317.31 1258.52	1298.03 1256.07
Symmetric/asymmetric stretching signals of the C-O-C bridge (glycosidic linkage)	1149.52 1060.79	1151.44 1064.65	1253.67 1062.72	1153.37 1066.58	1163.02 1062.44
C-O stretching vibration in secondary and primary OH groups	1026.08 985.57	1022.29 941.21	1018.36 985.89	1021.22 985.77	1004.86 968.37

**Table 7:** Crystallinity Index (%) of chitosans extracted with mineral acids (***Ch_HCl_*** and ***Ch_H2SO4_***) and organic acids (***Ch_Citric_***, ***Ch_Acetic_***, and ***Ch_Lactic_***).

Chitosan Samples	Mineral Acids		Organic Acids		
	*Ch_HCl_*	*Ch_H2SO4_*	*Ch_Citric_*	*Ch_Acetic_*	*Ch_Lactic_*
CrI	87.43%	82.61%	84.51%	82.02%	82.24%

## Data Availability

Not applicable.
